# Wait lists and adult general surgery: is there a socioeconomic dimension in Canada?

**DOI:** 10.1186/s12913-019-3981-9

**Published:** 2019-03-13

**Authors:** Jason M. Sutherland, Zuzanna Kurzawa, Ahmer Karimuddin, Katrina Duncan, Guiping Liu, Trafford Crump

**Affiliations:** 1 Centre for Health Services and Policy Research, School of Population and Public Health, University of British Columbia, 201-2206 East Mall, Vancouver, British Columbia V6T 1Z3 Canada; 2 Section of Colorectal Surgery, Department of Surgery, University of British Columbia, Vancouver, Canada; 3 Department of Surgery, University of British Columbia, Vancouver, Canada; 4 Department of Surgery, Cumming School of Medicine, University of Calgary, Calgary, Canada

**Keywords:** Wait times, Socioeconomic status, Access, General surgery

## Abstract

**Background:**

Little is known about whether patients’ socioeconomic status influences their access to elective general surgery in Canada. The purpose of this study was to assess the association between socioeconomic status and wait times for elective general surgery.

**Methods:**

Analysis of prospectively recruited participants’ data. The setting was six hospitals in the Vancouver Coastal Health Authority, a geographically defined region that includes Vancouver, British Columbia, Canada. Participants had elective general surgery between October 2013 and April 2017, community dwelling, aged 19 years or older and could complete survey forms. The outcome measure was wait time, defined as the number of weeks between being registered for elective general surgery and surgery date.

**Results:**

One thousand three hundred twenty elective general surgery participants were included in the study. The response rate among eligible patients was 53%. Regression analyses found no statistically significant association between patients’ wait time with SES, adjusting for health status, cancer status, surgical priority level, comorbidity burden and demographic characteristics.

Participants with proven or suspected cancer status had shorter waits relative to participants waiting for surgery for benign conditions. Participants with at least one comorbidity tended to experience shorter waits of approximately 5 weeks (*p* < 0.01). Pre-operative pain or depression/anxiety were not associated with shorter wait times.

**Conclusions:**

Although this study found no relationship between SES and surgical wait time for elective general surgeries in the study hospitals, patients in lower SES categories reported worse health when assigned to the surgical queue.

## Background

As wait times for elective surgery continue to be a prominent policy issue in publicly funded hospital insurance programs, such as in Canada’s provinces, equitable access to hospital care requires careful examination. In Canada, as elsewhere, non-emergent elective surgery patients access their treatment through a ‘first-in, first-out’ protocol based on their diagnosis [1]. However, surgeons have autonomy to re-order their wait lists to accommodate patients’ perceived urgency, symptom burden, or risk of adverse events. Little is known about whether socioeconomic status (SES), an individual’s or group’s relative position within society [2], influences surgeons’ decision-making relative to the duration that patients wait for elective (planned) surgery in Canada.

Two studies have investigated the relationship between access to elective surgery and SES in Canada; for 22 common surgeries, there was little evidence associating regional measures of SES with longer wait times [3]. While these studies provide an important view of equal access in Canada, they did not adjust for potential confounding by health status, cancer diagnosis and patient comorbidities [3, 4]. The exclusion of these potential confounders in other studies limits their generalizability, as there is evidence showing that patients in lower SES strata are more likely to delay treatment, present with more complicated cases and have been treated through emergency general surgery [5–7].

Comparing Canadian provinces with the United States’ performance on access to elective surgery is challenging; differences are primarily attributable to insurance coverage and comprehensiveness. Evidence from Australia, did find evidence of prioritization of higher SES patients not attributable to clinical or geographic differences in access [8]. Elsewhere, however, the findings are mixed [9]. A nine-country study of elderly patients in Europe found that high educational achievement was associated with shorter waits for specialist consultations and elective surgery. The income effect however was modest and varied. In Germany, a ten thousand Euro increase in income reduced wait times for specialists by 8%, while in Greece, the income effect reduced wait times for elective surgery by 26%. Conversely, the income effect in Sweden was associated with increased waits for surgery of 11% [10]. Other European studies have reported no association; in Norway, gender and SES were not associated with variation in wait times across seven surgical specialties [11]. The heterogeneity of findings provides a cautionary tale that even countries with population-based hospital insurance program have not been immune to inequities in access.

The mechanisms through which SES may be associated with patients’ wait for elective surgery are complex. Applying Andersen and Newman’s framework of health services use [12] provides insight regarding how these mechanisms could operate. Under this framework, health services use is a function of three factors including 1) predisposing factors, which are an individual’s socio-cultural characteristics; 2) enabling factors, which affect the logistics of obtaining care, such as having the financial or social support, or access to health providers and facilities, and, 3) need factors, such as how an individual and health care provider perceive or evaluate need.

Applied to the context of accessing elective surgery in Canadian provinces, a relevant socio-cultural characteristic may be expressed through educational attainment, whereby highly educated individuals may possess more health literacy [13], be more likely to identify surgeons with shorter wait times, or be more effective self-advocates [10]. The role of enabling factors is unclear; Canadian studies have reported that low SES patients visit GPs more than high SES patients [14, 15]. Lastly, surgeon perception of patients may introduce unconscious biases in prioritization – in a relevant study of general surgeons, factors associated with patient need were based on diagnosis, treatment options, patient characteristics, symptomatology and complications from the disease, risk of future complications, patient quality of life, psychosocial disease impact and logistic factors [16]. While SES was not listed among the factors, SES may play a role in shaping surgeons’ perceptions of symptomatology, quality of life and disease impact, though this research needs more development.

The objective of this study was to assess the association between SES and elective general surgery wait times, adjusting for potential confounders absent in many Canadian studies of access to care. The findings provide a modern interpretation of factors associated with waits for elective surgery. A null result would provide evidence that surgical resources, within the envelope of funding of elective general surgery, are being equitably allocated among the SES stratum. The issue of unequal access to elective surgery is pertinent in Canada, as waits can be long [17] and Canada routinely ranks at the bottom of international surveys for access to specialist care [18, 19].

## Methods

### Recruitment protocol

This study was based on a retrospective analysis of a sample of prospectively recruited elective general surgery patients in the Vancouver Coastal Health (VCH) Authority, a geographically defined region that includes Vancouver, British Columbia (BC). VCH services 25% of BC’s population of over one million residents. A convenience sample of fourteen general and colorectal surgeons in six hospitals agreed to have the population of their elective general surgery patients contacted to participate in the study.

Participants in this study were identified from the VCH Surgical Patient Registry, or wait list. The wait list comprises all surgical patients who consented for elective surgery in the region’s acute care hospitals. Participants had to be community-dwelling, 19 years or older, scheduled for surgery at least 7 days from being enrolled on the wait list, and able to respond to questions in English. All participants completed a survey package either online or by mail, which included a list of study personnel, stamped return envelope for mailed surveys, and a number of patient-reported outcome (PRO) measures assessing pre-operative health status and condition-specific quality of life.

The duration of each participant’s wait was extracted from the hospital records, and measured as the number of weeks between being registered on VCH’s wait list queue and surgery date. For this study, participants were recruited and returned their surveys between October 2013 and April 2017.

As surgeons can prioritize patients’ surgeries based on perceived severity, burden of illness, or risk of poorer post-operative outcomes, a measure of health status was needed to adjust wait times for potential confounding of health status. Participants completed the Euro-QoL’s EQ-5D(3 L), an instrument measuring general health status in five domains [20]. The instrument measures mobility, self-care, usual activities, pain/discomfort, and anxiety/depression. Participants score each item on three levels: no problem, some problems, and severe problems. Indicators were created for those reporting some or severe problems in the each of the five domains.

Participants’ PROs were linked with hospitals’ discharge summaries to identify participant characteristics, including sex, age, and date of surgery. Based on comorbidities reported in hospitals’ discharge summaries, participants’ comorbidity profile was reflected by the Charlson comorbidity index, an ordinal variable which represented the gradient of morbidity burden [21]. Examining the distribution of participants’ Charlson comorbidity index, values were categorized into values of zero, one, and two or more; sensitivity analyses revealed no impact of this categorization.

Cancer treatment is prioritized, with positive status often being treated in less than 4 weeks. Participants’ cancer status is noted when surgery scheduling occurs and is categorized as: proven, suspected, and not suspected. Cancer status was included in the model to reflect potential differences in wait times attributable to cancer status.

In BC, elective surgery patients are assigned a priority level, based on diagnosis, which represents the expected duration patients wait for their surgery. This five-level variable functions as a triage mechanism for prioritizing elective surgery; Priority 1 corresponds to highest urgency with a target wait period of 2 weeks, while Priority 5 corresponds to a target of 26 weeks. This variable was included in the analyses to adjust for the distribution of diagnoses across SES categories.

Socioeconomic status was represented by a five-level ordinal variable reflecting neighborhood-level information such as highest educational achievement, unemployment, income, and housing. Construction of the SES category was based on population-level census data independent from this study [22]. The first quintile represents neighborhoods in with the highest SES category, and the fifth quintile represents the lowest SES category. The geographically-defined variable was calculated at the level of dissemination areas, each representing between 120 and 140 households and between 400 and 700 persons.

The VCH Legal and Privacy Office completed a Privacy Impact Assessment to ensure the protocol was consistent with privacy legislation and patient information was adequately secured. The University of British Columbia’s Behavioural Research Ethics Board approved the study.

### Analysis

Summary statistics were generated to characterize the demographic categories of participants. Then, a linear regression was performed to identify factors associated with wait times. In the regression model, the dependent variable was wait time (weeks), while the independent variables included SES category, health status, cancer status, Charlson comorbidity index, priority level, sex and age categories. Model fit was evaluated using the AIC statistic. Given the skewed distribution of wait times, for sensitivity, a model with log-transformed wait times was tested, followed by a negative-binomial regression for over dispersion. In addition, a model with an interaction between SES category and priority level was explored.

There was a small amount of missing data. Multiple imputation was conducted and data were assumed missing at random. Two hundred imputations were used. To guide the final model selection, model fit was compared using the AIC statistic. All analyses were conducted using SAS 9.4 (Cary, NC).

## Results

The participation rate among eligible participants was 53%, resulting in a study sample of 1320 participants scheduled for planned (elective) general surgery. Participants were approximately 4 years older than non-participants; no differences were detected in participation rates between sexes. Other potential confounding factors were not observable among non-participants.

As shown in Table 1, the levels of SES were approximately equally represented among participants; the highest SES category was the most prevalent (21.4%) and the least prevalent was the lowest SES category (17.0%). Slightly more than half of respondents were male (54.5%) and the mean age was 60.5 years. One fifth of the sample were scheduled for surgery for a diagnosis of cancer, and one third had at least one comorbid condition.

**Table 1:** Characteristics of the sample of general and colorectal surgery participants

	Number	Percent
SES Category		
1 (Highest SES)	282	21.36
2	237	17.95
3	246	18.64
4	226	17.12
5 (Lowest SES)	224	16.87
Sex		
Female	601	45.53
Male	719	54.47
Age		
Mean (standard deviation)	60.52	(13.61)
Cancer		
Not suspected	906	68.95
Suspected	285	21.69
Proven	123	9.36
Charlson comorbidity index		
0	866	65.61
1	91	6.89
2+	363	27.50
Priority levels (Target weeks)		
1	47	3.56
2	393	29.77
3	157	11.89
4	425	32.20
5	298	22.58
5 Most common procedures		
Abdominal colorectal surgery	483	36.59
Hernia surgery (non-incisional)	244	18.48
Anorectal surgery	234	17.73
Gastric bypass	167	12.66
Cholecystectomy	126	9.55
EQ5D (Reporting some or severe problems before surgery)		
Mobility	313	24.21
Self-care	94	7.26
Usual activities	477	36.86
Pain/discomfort	714	55.22
Anxiety/depression	468	36.17

Most patients were undergoing abdominal colorectal surgery (36.6%) followed by non-incisional hernia surgery (18.5%). Rates of missing data were low; cancer status was missing for less than 0.5% of participants, and items of the EQ-5D(3 L) were missing for less than 2.0% of participants.

As shown in Table 2, the average wait time was over 11 weeks. Summarizing patient-reported health status in Table 3 found that a majority of the sample reported some or severe problems with pain/discomfort (55.2%) and over one third of the sample reported problems with anxiety/depression or performing usual activities. Those in the lowest SES category reported the most problems with pre-operative self-care, usual activities, pain/discomfort, and anxiety/depression compared to the other categories (Table 3).

**Table 2:** Wait times (weeks) for general and colorectal surgery across SES categories

	Mean	Standard Deviation	Median	Range	
All	11.43	11.71	6.64	0.86	87.14
SES Category					
1 (Highest SES)	11.79	11.45	6.93	1.29	54.29
2	11.23	10.93	6.86	0.86	64.14
3	11.37	11.78	6.57	1.14	67.00
4	11.78	12.37	7.00	1.29	87.14
5 (Lowest SES)	10.73	11.67	6.00	1.29	64.71

**Table 3:** Percent of patients reporting some/severe problems before surgery across SES categories

	Mobility	Self-care	Usual activities	Pain/discomfort	Anxiety/depression
All	24.21	7.26	36.86	55.22	36.17
SES Category					
1 (Highest SES)	17.09	4.36	30.55	50.36	30.55
2	24.46	5.13	35.62	55.56	35.04
3	23.05	8.23	36.63	54.96	36.21
4	26.91	8.04	35.27	54.91	36.32
5 (Lowest SES)	26.85	10.19	45.83	57.87	39.81
Chisq (*p*-value)	0.06	0.08	0.01	0.55	0.30

The multivariable analyses of wait times shown in Table 4 found no statistically significant differences between wait times and levels of SES, adjusting for potential confounders described above. Male sex was associated with a longer wait time (*p* < 0.01), although the difference is likely not clinically significant. The results were insensitive to log transformation of wait times and negative-binomial regression; results from the linear regression with untransformed wait times are reported as they are easiest to interpret. The best fitting model included the priority level variable.

**Table 4:** Regression results modeling relationship between SES and wait time (weeks)

Model Effect	Estimate	Standard Error	*P*-value
Intercept	12.982	1.599	<.0001
SES Category			
1 (Highest SES)	Ref.		
2	−0.218	0.920	0.813
3	0.214	0.911	0.814
4	0.513	0.932	0.582
5 (Lowest SES)	−0.094	0.943	0.920
Sex			
Female	Ref.		
Male	2.126	0.600	0.000
Age			
	0.029	0.023	0.216
Cancer			
Not suspected	Ref.		
Suspected	−4.640	1.261	0.000
Proven	−2.586	1.160	0.026
EQ5D Mobility			
None	Ref.		
Some/severe problems	−1.902	0.864	0.028
EQ5D Self-Care			
None	Ref.		
Some/severe problems	−2.301	1.245	0.065
EQ5D Usual activities			
None	Ref.		
Some/severe problems	−0.940	0.765	0.220
EQ5D Pain/discomfort			
None	Ref.		
Some/severe problems	0.086	0.706	0.903
EQ5D Anxiety/depression			
None	Ref.		
Some/severe problems	0.098	0.652	0.880
Charlson comorbidity			
0	Ref.		
1	−4.795	1.166	<.0001
2+	−5.373	0.991	<.0001
Priority levels			
1	−1.058	1.701	0.534
2	−4.165	1.189	0.001
3	0.226	1.067	0.832
4	1.360	0.815	0.095
5	Ref.		

Participants with proven or suspected cancer status had shorter waits, on average, of between 2.6 and 4.6 weeks, respectively, relative to participants waiting for elective surgery for benign conditions. Participants with at least one comorbidity tended to experience shorter waits of about 5 weeks (*p* < 0.01). Pre-operative pain or depression/anxiety were not associated with shorter wait times. An interaction between priority level and SES was not significant and did not improve model fit (results not shown).

## Discussion

This study found no evidence between patients’ socioeconomic stratum with wait time for elective general surgery, adjusting for health status and other observable patient factors. Even upon inclusion of additional covariates such as self-reported health and comorbidities, these results are consistent with prior, albeit very limited, Canadian experience [3, 4, 23, 24]. This study found that participants in lower SES quintiles self-reported poorer health, describing more problems with mobility, self-care, usual activities, pain/discomfort and anxiety/depression at the time surgery was booked, echoing past studies [25, 26]. This finding may signal that while there is ‘equality of outcome’ among SES groups with respect to wait times, there are still potential inequities during the wait time.

The setting of this study, BC, has first-dollar (no cost to the patient) coverage for accessing hospital care through the provincial health insurance program. However, the population-based insurance program does not extend to non-hospital based drugs, allied therapies or psychosocial counselling, which are paid through out-of-pocket by patients or through workplace-based health insurance.

While this study demonstrated equity in access, participants in the lowest SES face unequal economic pressures associated with their condition. Owing to the hospital- and physician-focused health insurance plan, participants are exposed to out-of-pocket medical expenses attributable to their condition during their wait [27, 28]. Patients in low SES categories are less likely to have deep financial resources or employer-based insurance, potentially inducing disparities in financial stress.

While the financial resources of participants were unmeasured in this study, a Canadian study reported that pain management utilization increased while patients waited for gynecological surgery [29], providing some evidence that equal access to elective surgery may manifest as disproportionate financial or psychological stress on some patients in lower SES categories during their wait for elective surgery. Other countries’ health insurance program that provide more comprehensive health insurance coverage, such as Norway or the National Health Service (United Kingdom) [30], may have a more robust capacity for assisting patients manage their non-surgical related domains of health, such as pain or anxiety, during their wait time by paying for drugs, therapies and psychosocial counseling.

For Canada’s provinces, and other countries whose health insurance programs do not extend to all medical expenditures, there are opportunities for improving patients’ in the lowest SES categories health status during the wait. Improvements could take the form of subsidizing costs related to their underlying condition during their wait for surgery. Future research could examine the differential use of therapies for management of conditions during the wait time across SES strata.

For such policies to be actionable, however, clinicians and hospital administrators would require unbiased patient-level indicators of patients’ SES. In some US cities, neighborhood level indicators have been used to flag patients at risk of medication non-adherence and higher risk of increased utilization [31]. However in the Canadian context, appending this type of data is novel.

Alternatively, surgical wait lists could be managed to prioritize improvements of patients’ quality of life. Survey results of the general population and healthcare providers have found support for prioritization of surgical wait lists based on the intensity of patients’ suffering [32]. One caveat is that although studies have found general surgeons incorporate quality of life into their waitlist prioritization [16], they are rarely trained to do so [33]. One study found that there was low degree of agreement in prioritizing wait-times of standardized patients among surgeons [33], suggesting that by providing surgeons with training in quality of life assessment, more equitable access to health could be achieved.

While this study focuses on elective general surgery in a populous and diverse region of Canada, the process for accessing elective surgery is similar among specialties. These results may hold across Canada; all provinces have a patchwork of insurance coverage for non-hospital based drugs, allied therapies or non-hospital based mental health services. Even though access appears equitable, additional research is needed to reduce financial disparities faced by surgical patients. Nevertheless, given mixed evidence internationally, this study recommends for similar analyses to be conducted in other settings.

Additional limitations to the generalizability of this study should be noted. An area-level variable was used as a proxy for individual-level SES. Although this approach is common, the method does introduce uncertainty into the findings consistent with this study’s inability to ascertain patients’ SES. Despite this, there was a gradient in participants’ health when moving from high SES to low SES consistent with existing evidence, suggesting the area-level indicator was sensitive to SES. Furthermore, as this study used a non-random sample of surgeons, there is potential selection bias; however the study did not have access to detailed surgeons’ characteristics, such as training or treatment preferences, to enable comparison with non-participating surgeons.

While all eligible patients consenting to elective surgery in VCH were contacted to participate and there was sufficient variation among SES categories in the analysis, there may have been unobservable differences between participants and non-participants. Evidence suggests that those with lower SES and limited access to insurance are more likely to cancel or no-show for an appointment [34]. While accessible data in this study’s jurisdiction does not provide a reason for surgery cancellation, this study observed a roughly equally distributed proportion of participants across SES categories, meaning those with lower SES may not have been underrepresented due to cancellations/no-shows and providing some assurance on representativeness. Future investigation of the association between waiting times and cancellations/no-shows across SES strata would complement this study’s findings. While this study found no association between SES and wait times on the surgical queue, data regarding the time between referral and specialist consultation is not standardized nor routinely collected in this jurisdiction. Future research should focus on this earlier delayed access to consultation. The response rate in this study was similar to other studies collecting PROs from general and colorectal patients (30–75%) [35–37]; though this study applied evidenced-based strategies to bolster recruitment and retention, including reminder emails/calls, remailing surveys, and adding headers/teasers to communications [38], efforts to limit non-response bias should continue to be a priority. Acknowledging this, the *p*-values were very large, and it is conceptually unclear what potential differences between responders and non-responders could change the outcome of the analysis.

## Conclusions

This study found no relationship between SES and surgical wait time for elective general and colorectal surgeries in VCH hospitals. Centrally supported efforts to incorporate SES into surgical prioritization may improve health status among surgical patients in the lowest SES categories.
